# How biopsychosocial depressive risk shapes behavioral and neural responses to social evaluation in adolescence

**DOI:** 10.1002/brb3.2005

**Published:** 2021-03-04

**Authors:** Jason Stretton, Nicholas D Walsh, Dean Mobbs, Susanne Schweizer, Anne‐Laura van Harmelen, Michael Lombardo, Ian Goodyer, Tim Dalgleish

**Affiliations:** ^1^ Medical Research Council Cognition and Brain Sciences Unit University of Cambridge Cambridge UK; ^2^ School of Psychology Faculty of Social Sciences University of East Anglia Norwich UK; ^3^ Division of Humanities and Social Sciences California Institute of Technology Pasadena CA USA; ^4^ Division of Psychology and Language Sciences University College London London UK; ^5^ Developmental Psychiatry Section Department of Psychiatry University of Cambridge Cambridge UK; ^6^ Department of Psychology and Center for Applied Neuroscience University of Cyprus Nicosia Cyprus; ^7^ Cambridgeshire and Peterborough NHS Foundation Trust Cambridge UK

**Keywords:** adolescence, biopsychosocial, depressive risk, emotion context insensitivity, partial least squares

## Abstract

**Introduction:**

Understanding the emotional responsivity style and neurocognitive profiles of depression‐related processes in at‐risk youth may be helpful in revealing those most likely to develop affective disorders. However, the multiplicity of biopsychosocial risk factors makes it difficult to disentangle unique and combined effects at a neurobiological level.

**Methods:**

In a population‐derived sample of 56 older adolescents (aged 17–20), we adopted partial least squares regression and correlation models to explore the relationships between multivariate biopsychosocial risks for later depression, emotional response style, and fMRI activity, to rejecting and inclusive social feedback.

**Results:**

Behaviorally, higher depressive risk was associated with both reduced negative affect following negative social feedback and reduced positive affect following positive social feedback. In response to both cues of rejection *and* inclusion, we observed a general neural pattern of increased cingulate, temporal, and striatal activity in the brain. Secondly, in response to *rejection only*, we observed a pattern of activity in ostensibly executive control‐ and emotion regulation‐related brain regions encompassing fronto‐parietal brain networks including the angular gyrus.

**Conclusion:**

The results suggest that risk for depression is associated with a pervasive emotional insensitivity in the face of positive and negative social feedback.

## Graphical abstract

To understand the emotional responsivity style and neurocognitive profiles of depression‐related processes in at‐risk youth may be helpful in predicting those most likely to develop affective disorders. We explored the relationships between multivariate biopsychosocial risks for later depression, emotional response style and fMRI activity, to rejecting and inclusive social feedback. Data indicate risk for depression in adolescence is associated with a pervasive emotional insensitivity in the face of positive and negative social feedback.

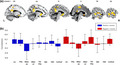

## 1 INTRODUCTION

With an estimated 300 million people suffering from depression, major depressive disorder (MDD) is the leading cause of disability globally (WHO, 2017). Many cases of MDD onset prior to or during adolescence (Kessler et al., 2005), and while there is a growing body of evidence surrounding the epidemiology of childhood and adolescent depression, it is still difficult to predict who will go on to develop MDD (Hankin, 2015). Research into etiological mechanisms needs to investigate precursors of MDD within the context of wider socioemotional development. In this study, we explored the relationship between biopsychosocial risk for depression and affective and neural responses to positive and negative social evaluation.

### 1.1 Emotional responsivity in depression

MDD is primarily characterized as a disorder of mood, indexed by alterations in the style of emotional responding to positive and negative stimuli (Rottenberg et al., 2005). There are several competing accounts as to how the depressed mood state impacts emotional responding. Negative potentiation views propose that those with MDD show increased negative reactions to negative stimuli, with negligible differences in positive reactivity, relative to nondepressed peers (Golin et al., 1977). Positive attenuation views, in contrast, propose reduced positive reactivity to positive stimuli in those with MDD, with little or no differences in negative reactivity (Allen et al., 1999), though as this view focuses primarily on reactivity to positive stimuli, positive attenuation is compatible with negative potentiation (i.e., individuals with MDD could exhibit both patterns simultaneously) (Bylsma et al., 2008). Finally, the emotion context insensitivity (ECI) hypothesis (Rottenberg et al., 2005) proposes *reduced* emotional reactivity to both positive and negative stimuli, in line with evolutionary accounts of depression as a functional state that fosters motivational disengagement from the environment (Allen & Badcock, 2003; Beck & Bredemeier, 2016; Gilbert & Allan, 1998; Nesse, 2000). Meta‐analysis of studies evaluating emotional reactivity in MDD suggests that the ECI account is most consistent with the data, with clinically depressed individuals exhibiting reductions in both positive and negative affect relative to nondepressed peers (Bylsma et al., 2008). Furthermore, formerly depressed individuals who are currently not experiencing a depressive episode have been shown to exhibit an ECI style of emotional responding relative to never‐depressed controls, consistent with the notion that ECI may reflect a trait‐like depressive disposition (Iacono et al., 1984). Consistent with this, in such remitted samples, degree of ECI has been shown to predict later depressive relapse (Lethbridge & Allen, 2008). This raises the important question as to whether those who have never experienced depression but who are deemed at risk of later MDD onset would exhibit systematic differences in the way that they respond to emotional provocation and whether these differences would be consistent with an ECI analysis.

### 1.2 Depression risk

There are multiple pathways to depression, with risk factors spanning the entire biopsychosocial spectrum including: (a) childhood adversity (CA)—comprising diverse environmental factors, including but not limited to physical abuse, sexual abuse, emotional maltreatment, low socioeconomic status, parental psychopathology, negative life events, and family discord. CA is a robust predictor of later psychopathology including depression (Costello et al., 2005; Spinhoven et al., 2010; van Harmelen et al., 2016); (b) predisposing biological factors including increased cortisol reactivity (Bruce et al., 2009; Power et al., 2012); (c) clinical predictors including presence of subclinical levels of depressive symptomatology, a history of previous psychiatric difficulties (Kim‐Cohen et al., 2003), frequency of mild daily stressors (Monroe & Harkness, 2005), and psychological factors such as high neuroticism (Clark et al., 1994) and low self‐esteem (Orth et al., 2008). This constellation of risk across the lifespan has been framed within a triple vulnerability model (Barlow, 2000) which proposes three strata of risk—general biological and subsequent early environmental (predominantly CA) vulnerabilities that then provide a diathesis context for later stressors such as negative life events, social isolation, and other sources of distress that are disorder‐specific and in this case lead to the onset of depression (Brown & Naragon‐Gainey, 2013).

This extensive constellation of identified biopsychosocial risk factors comprises elements that are arguably highly interrelated, making it difficult to disentangle unique and combined effects at a biological level (Rutter, 2012). The current literature also invariably focuses on one or a small number of risk factors. Consequently, there is a need for multivariate approaches encompassing multiple risk factors to better inform the relationship between depression risk and the socioemotional and neurocognitive profiles of ecologically valid emotional responding. In the present study, we adopted a multivariate approach to risk, including a range of key biological, social, and psychiatric variables that had been evaluated as part of a longitudinal cohort study of adolescent emotional development (Goodyer et al., 2010). We used a social evaluation paradigm (Dalgleish et al., 2017) to examine the relationship between biopsychosocial risk for depression, and emotional responses to positive and negative information, in this case rejecting and inclusive social feedback which we reasoned would have particular emotional potency for an adolescent sample (Blakemore, 2008).

Investigating the association between risk for depression and emotional responses to social evaluation is critical as it is a period of rapidly changing social environments, and the need to belong is strong and important to fulfil (Patrick et al., 2007). Cues of social acceptance and social rejection provide critical information about the adolescent's degree of inclusivity at any given social moment (Baumeister & Leary, 1995). A social evaluation paradigm therefore represents a compelling context within which to examine patterns of emotional responsivity in this age group. Prior studies show blunted positive affect is evident in children at high risk for developing depression due to parental psychopathology (Weissman et al., 1987) and lower levels of positive emotion in nonclinical adolescent samples can predict depressive symptoms a year later (Lonigan et al., 2003). Furthermore, adolescents at high risk for developing depression and currently depressed adolescents display similarly decreased positive affect compared with low‐risk adolescents (Dietz et al., 2008). Based on this, and the aforementioned meta‐analytic evidence in depressed adults (Bylsma et al., 2008), our behavioral hypothesis was adolescents with higher levels of multivariate risk for later depression would exhibit ECI in their emotional responses, with reduced positive and negative reactivity to cues of social inclusion and rejection, respectively.

### 1.3 Depression risk and neural responsivity to psychosocial stress and reward

As well as the relationship between multivariate depression risk and behavioral indices of emotional reactivity in response to social rejection and inclusion, we additionally wanted to elucidate patterns of neural activity. Specifically, we planned to elucidate latent brain‐behavior relationships using a multivariate partial least squares (PLS) correlation approach (Krishnan et al., 2011) to investigate the latent structure of the biopsychosocial factor(s) associated with risk and their relationship with neural activity during the social evaluation task measured using functional magnetic resonance imaging (fMRI).

Differential patterns of neural processing of stress and reward are well‐established in the MDD literature (see (Pizzagalli, 2014) for review), indicating that the ventral and dorsal striatum may play a pivotal role. The processing of monetary reward (Pizzagalli et al., 2009) and socially appetitive stimuli (Elliott et al., 1998; Epstein et al., 2006) in MDD has been consistently associated with blunted activation of the ventral and dorsal striatum thought to reflect dysfunction in coding the motivational significance of rewards and deficiencies in positive‐reinforcement learning, respectively (Pizzagalli, 2014).. This is in line with event‐related potential (ERP) data which consistently shows diminished activity to the processing of motivationally salient stimuli and to the receipt of reward, suggesting depression is associated with emotional disengagement and deficits in reward processing (Proudfit et al., 2015).

In addition, there is evidence for disrupted processing of psychosocial stress and reward in samples defined by risk factors for depression and for psychopathology more generally. In the context of CA psychosocial stress in the form of social rejection has been associated with increased dorsomedial prefrontal cortex (PFC) activation in young adults with a history of childhood emotional maltreatment (van Harmelen et al., 2014) and with reduced connectivity and activation of the dorsal anterior cingulate cortex (dACC) and dorsolateral PFC in children exposed to neglect, physical abuse and domestic violence (Puetz et al., 2014). Additionally, increased subgenual PFC activity during social rejection has been shown to be predictive of depressive symptomatology one year after assessment (Masten et al., 2011). Only one preliminary study has investigated the psychosocial reward of social acceptance, showing that high‐risk youth, defined as those with a parental history of depression, exhibited reduced responses to acceptance in the caudate, insula, and ACC and increased activity in fronto‐temporal regions relative to low‐risk controls (Olino et al., 2015). This is in line with monetary reward tasks, which have generally shown a reduced striatal response to reward anticipation and feedback in those who have experienced early adversity (Goff et al., 2013; Hanson et al., 2016; Mehta et al., 2010). Taken together, these results suggest the striatum may be a potential neural substrate for the interaction between stress, reward, risk, and MDD (Pizzagalli, 2014). Our social evaluation paradigm provides context for both psychosocial reward, through social inclusion, and psychosocial stress, through social rejection and is thus well suited to investigate this further.

There are a number of possible ways in which patterns of neural activity across these implicated circuits associated with positive and negative social feedback may relate to the different theories articulated above concerning emotional responsivity in depression and in those at risk. First, attenuated emotional responsiveness may be reflected in differential activity in emotion regulation and executive control brain regions in the context of either just positive (the positive attenuation model) or both positive and negative (the ECI model) social feedback, reflecting enhanced top‐down control over emotional responsiveness within these neural circuits. Secondly, there may be an analogous shift in the influence of bottom‐up processes whereby activity in reward‐related brain regions might be altered in response to positive evaluations, relative to neutral evaluations (positive attenuation models). Finally, activity in limbic brain regions predominantly associated with negative emotionality, such as the amygdala, may also be affected (negative reactivity and ECI models) in response to negative evaluations relative to neutral evaluations. Examination of patterns of neural activity using FMRI during the social evaluation task will enable us to investigate these different possibilities.

## 2 MATERIALS AND METHODS

### 2.1 Participants

Participants [*N* = 56; Mean (*SD*) age = 18 (0.7), range 17–20 years; 31 females, see Table 1] were a subset from the ROOTS study (Total *N* = 1,143), a population‐derived longitudinal investigation of adolescent emotional development (Goodyer et al., 2010). Inclusion criteria for this neuroimaging sub‐study were as follows: normal or corrected‐to‐normal vision; English speaking; and of Northern European descent (to facilitate genetic allele comparisons for different components of the study). Exclusion criteria were as follows: any history of neurological trauma resulting in loss of consciousness; current psychotropic medication use; current neurological disorder; current DSM‐IV Axis 1 disorder; presence of metal in body; specific learning disability, and IQ < 85 on the Weschler Abbreviated Scale of Intelligence (WASI). The selection and recruitment process sought to recruit a subsample with a broad range of depressive risk and is described in more detail in (Walsh et al., 2012) (see also Figure S1).

**TABLE 1:** Sample demographics and social evaluation ratings

	*N* = 56
Age [Years (*SD*)]	18 (0.7)
Gender [m/f]	25/31
IQ [Mean(*SD*]	107 (9.4)
SES (ACORN) [*N*/%]	
Wealthy Urban	32 (57%)
Comfortable	14 (25%)
Moderate means/hard pressed	9 (18%)
CA (±)	26/30
Psychiatric history (±)	18/38
Parental psychiatric history (±)	30/26
Positive affective response rating	2.05 (1.22)
Negative affective response rating	−3.14 (1.73)

Note

_Means and standard deviations () are shown for age, positive affective response ratings, and negative affective response ratings. ACORN; A Classification of Residential Neighborhoods; CA, childhood adversity; SES; Socioeconomic status._

Importantly, participants recruited to this neuroimaging sub‐study showed no significant selection bias compared with the total ROOTS sample in terms of gender ratio or socioeconomic status as assessed using the UK ACORN (A Classification Of Residential Neighborhoods) geodemographic measure (Morgan & Chinn, 1983) (http://www.caci.co.uk). Participants in the present study did have lower levels of self‐reported depressive symptoms at the time of scanning relative to the overall ROOTS sample (measured age 17), due to the exclusion of those ROOTS members with current psychiatric disorders from the present study (see below).

### 2.2 Ethical considerations

The study was carried out in accordance with the Declaration of Helsinki and Good Clinical Practice guidelines and approved by the Cambridgeshire Research Ethics Committee. All participants provided written informed consent. If participants were under 18 years old, informed written consent was gathered from a parent and/or legal guardian.

### 2.3 Biopsychosocial risk variables

The biopsychosocial risk variables for depression assessed in the current study were collected longitudinally at the following time‐points: Time 1 aged 14; Time 2 aged 16; Time 3 aged 17. Assessed variables included: retrospectively reported childhood adversities (CA; Time 1); previous participant psychiatric history (Times 1 and 3); parental psychiatric history (Time 1); family discord as measured by the Family Assessment Device (FAD; Time 1); recent negative life events assessed at age 14 (RNLE14; Time 1) and again at age 17 (RNLE17; Time 3); morning cortisol levels (Time 1); and current depressive symptomatology (on the Beck Depression Inventory; BDI; Time 3). Details about each variable are included in the Data S1, Figure S2, and Table S2. See Table S3 for a correlation matrix of the variables.

### 2.4 Social evaluation task

The social evaluation task was administered after Time 3. A full task description can be found in the Data S1 and in (Dalgleish et al., 2017). The task is designed to elicit affect based on peer‐mediated social feedback based on personally salient information. Briefly, each participant is subject to 36 judgements—evaluations by 6 judges on 6 social attributes—based on a pre‐recorded video of the participant discussing their lifetime aspirations. The six social attributes comprise social competence, motivation, self‐confidence, personal strength, social attractiveness, and emotional sensitivity. Judgements are delivered as part of a “Big Brother” style voting assessment of the participant, relative to three peers. The task is delivered within the MRI scanner. Each of the 36 judgement epochs begins with an 8‐s slide showing which judge would be judging which attribute (e.g., *David* will now be judging you on *social attractiveness*). This is followed by an 8‐s fixation period, and then an 8‐s result (feedback) slide, showing whether each participant has been judged to be the best (positive feedback), middle (neutral feedback), or worst (negative feedback) on that particular attribute relative to their peers. Following each result slide, participants complete a 10‐s VAS affective response rating (ranging from 0 (extremely negative)—11 (extremely positive)) to index how they feel following the feedback. The peers and judges were in fact fictional and the 36 trials were rigged so as to provide 12 trials of “best” feedback (Positive trials), 12 trials of “middle” feedback (Neutral trials), and 12 trials of “worst” feedback (Negative trials) for each participant. Attribute and judge order were counterbalanced across participants. At the end of the 36 feedback trials, an overall judgement from each judge was presented to the participant, detailing whether they had made it through to the next round. Of these six final judgements, 5 were “worst” and one was “middle” resulting in the subject being told they had been voted out. This was done to maintain task credibility, and these ratings were set aside from the analysis.

### 2.5 Behavioral analysis

PLS regression is a dimension reduction approach that is coupled with a regression model. PLS works well for data with relatively small sample sizes and a large number of parameters (Wold et al., 1984). The algorithm reduces the number of parameters using a technique similar to principal components analysis to extract a set of components that describes maximum correlation between the predictors and response variable(s). Components were evaluated for significance based on the percentage of variance explained in both the predictor variables and the response variable and were retained if they explained more than 10% (equivalent to small effect size (Cohen, 1992)) of the variance in both variable sets. If a component was retained, the factor loadings were then used to determine the importance of each variable to the component, measured as correlation coefficients ±0.4. Low factor loadings indicate relatively low importance to the projection of the latent variable but still contribute to the overall pattern of the latent factor. First, all psychiatric risk variables (CA, RNLE14, RNLE17, previous psychiatric history, parental psychiatric history, FAD score, BDI score, cortisol) were entered into separate PLS regression models to predict the mean affective response ratings across the Positive and Negative (each minus Neutral) trials of the social evaluation task, obtained during the fMRI session. All analyses were carried out using SPSS v22.

### 2.6 Image acquisition and preprocessing

MRI scanning was conducted at the Medical Research Council Cognition and Brain Sciences Unit on a 3‐Tesla Trio Tim Magnetic Resonance Imaging scanner (Siemens, Germany) by using a 32‐channel head coil gradient set. Whole‐brain data were acquired with echoplanar T2*‐weighted imaging (EPI), sensitive to BOLD signal contrast (48 sagittal slices, 3 mm‐thickness; TR = 2,000 ms; TE = 30 ms; flip angle = 78°; FOV 192 mm; voxel size: 3 × 3 × 3 mm). To provide for equilibration effects the first 5 volumes were discarded. T1‐weighted structural images were acquired at a resolution of 1x1x1 mm.

SPM8 software (www.fil.ion.ucl.ac.uk/spm/) was used for fMRI data analysis. The EPI images were sinc interpolated in time for correction of slice timing differences and realignment to the first scan by rigid body transformations to correct for head movements. The mean EPI for each subject was inspected after realignment to ensure there were none with signal dropout. Field maps were estimated from the phase difference between the images acquired at the short and long TE and unwrapped, employing the FieldMap toolbox. Field map and EPI imaging parameters were used to establish voxel displacements in the EPI image. Application of the inverse displacement to the EPI images served the correction of distortions. Utilizing linear and nonlinear transformations, and smoothing with a Gaussian kernel of full‐width‐half‐maximum (FWHM) 8‐mm, EPI, and structural images were coregistered and normalized to the T1 standard template in Montreal Neurological Institute (MNI) space (International Consortium for Brain Mapping). Global changes were removed by proportional scaling, and high‐pass temporal filtering with a cutoff of 128 s was used to remove low‐frequency drifts in signal.

### 2.7 Imaging analysis

Briefly, after preprocessing statistical analysis was performed using the general linear model. Analysis was carried out to establish each participant's voxel‐wise activation during the feedback and rating trials. Activated voxels in each experimental context were identified using an epoch‐related statistical model representing each of the three feedback trials and subsequent affect ratings, convolved with a canonical haemodynamic response function and mean‐corrected. Six head‐motion parameters defined by the realignment were added to the model as regressors of no interest. Multiple linear regression was then applied to generate parameter estimates for each regressor at every voxel. At the first level, the following feedback contrasts (based on activations to the result slides) were generated; “positive feedback” minus “neutral feedback” to isolate social acceptance/inclusion; “negative feedback” minus “neutral feedback” to isolate social rejection/exclusion.

### 2.8 Multivariate associations between biopsychosocial risk and fMRI activations

To identify neural systems correlated with a latent variable (LV) for biopsychosocial psychiatric risk, measured by our combination of CA, RNLE14, RNLE17, previous psychiatric history, parental psychiatric history, FAD score, BDI score, and morning cortisol, we applied the multivariate statistical technique of PLS correlation, using PLSGUI (http://www.rotman‐baycrest.on.ca/pls/). The goal of this “Behavioral PLS” is to take 2 multivariate matrices (one for behavioral variables and the other for brain variables) and find the combination of LVs from the brain and behavioral matrices that express the largest amount of common information (i.e., largest covariance) (Krishnan et al., 2011). This has been applied in studies of obsessive‐compulsive disorder, autism, and psychotic disorder (Dean et al., 2013; Ecker et al., 2012; Menzies et al., 2007) among others. In our case, these analyses identify the set of brain voxels most robustly correlated with the LV pattern underlying biopsychosocial risk measures in adolescents responding to social evaluation. A permutation test (10,000 permutations) evaluated the significance of identified LVs, and 10,000 bootstrap resamples were used to assess the reliability of voxels with the strongest contribution to the pattern. For visualization of the most reliable voxels contributing to the patterns, we used a bootstrap ratio of (−) 3 and a cluster extent threshold of 250 voxels. The bootstrap ratio can be viewed/interpreted as a pseudo *Z*‐statistic, since it is the ratio of a voxel's “salience” (i.e., a latent variable linear combination of the original variables) divided by the standard error estimated from bootstrapping (Krishnan et al., 2011). This bootstrap ratio allows us to infer which voxels were most important and reliable in terms of their contribution to the overall pattern identified by PLS.

Using this approach, we investigated whether our combination of adverse biopsychosocial variables was associated with activation patterns in each of the feedback contrasts. Guided by the whole‐sample conjunction analysis results reported previously (Dalgleish et al., 2017) and our hypothesis of a common pattern of heightened risk being associated with emotional attenuation for both Positive and Negative social feedback in the behavioral data, we initially ran a “two‐condition PLS” (Positive > Neutral and Negative > Neutral) to assess whether any latent brain‐behavior pairs explained a similar degree of variance across both feedback conditions. We then ran separate PLS analyses on the Positive > Neutral and Negative > Neutral contrasts in order to test for any context‐specific effects of positive and negative evaluation alone.

### 2.9 Analysis excluding participants with a psychiatric history

Although prior mental health diagnosis is a risk factor for future psychopathology, in the context of depression vulnerability and to clarify the relevance of our findings to those who had never previously met criteria for a diagnosis, it was important to test that any resulting brain‐behavior pairs were not specific to prior mental health difficulties. Thus, we ran an additional sensitivity analysis by conducting the same PLS analyses on the subsample of participants who had no previous psychiatric history of any kind (*n* = 38).

### 2.10 Univariate associations within the fMRI data

We also performed follow‐up univariate analyses in SPM8 (Wellcome Trust Centre for Neuroimaging, London, UK). These allow us to test the univariate contribution of each psychosocial risk variable to each feedback contrast in order to increase confidence in the contribution of any one single variable to patterns of neural activity. A series of 1‐sample *t* tests were run on each feedback contrast with the following risk variables as covariates: CA, RNLE14, RNLE17, previous psychiatric history, parental psychiatric history, FAD score, BDI score, and morning cortisol level. We performed a whole‐brain analysis, and images were assessed for cluster‐wise significance using a cluster‐defining threshold of *p* < .001 uncorrected; the .05 FWE‐corrected critical cluster size was 350 voxels (https://doi.org/10.5281/zenodo.1689891). In order to additionally test the contribution of each single variable to the patterns of activity across both positive and negative evaluation, we entered the Positive > Neutral and Negative > Neutral feedback contrasts into a series of repeated measures ANOVAs including the same covariates listed above.

## 3 RESULTS

### 3.1 Behavioral data

We have previously reported the main behavioral and neural effects across the whole sample on the Social Evaluation Task (Dalgleish et al., 2017) and the present focus is on the relationship between task performance and biopsychosocial risk for depression. To summarize these prior findings, as expected participants overall rated Negative social feedback as more upsetting than Neutral feedback (*t* = 12.6, *df* = 55, *p* < .001) and Positive feedback as less upsetting than Neutral (*t* = 13.5, *df* = 55, *p* < .001) (see Table 1).

Turning to the relationships involving biopsychosocial risk, our PLS regression model of the behavioral data identified one optimal risk component that predicted affective response ratings to Negative (minus Neutral) feedback trials. This component loaded most strongly on CA (0.55), BDI scores (0.44), and FAD scores (0.57) (see Table S4 for full component loadings). The component explained a small‐medium amount of variance (Cohen, 1992) in the predictor risk variables (*R* ^2^ = .16) and a medium amount of variance in the affective response ratings to these negative trials (*R* ^2^ = .23). A Pearson correlation confirmed that this risk component was associated with *lower levels* of participant‐rated negative affect (*r* = .48, *p* < .001; n.b. greater negative affect was indexed with increasing negative integers as per the subtraction formula to derive the rating score, hence the positive correlation). The predominantly positive loadings (6 of the 8 risk variables), together with an overall association with lower negative affect, are therefore in line with the ECI hypothesis of higher depression risk being associated with blunted emotional responding to negative feedback (Rottenberg et al., 2005), and counter to negative potentiation models relating risk to augmented responding (Golin et al., 1977; Rottenberg et al., 2005).

In predicting affective response ratings to Positive (minus Neutral) feedback, PLS again identified one risk component. This component loaded most strongly on CA (−0.44) and FAD scores (−0.65), as with the Negative feedback contrast, as well as morning cortisol (−0.41), and explained a small‐medium amount of variance in the predictor risk variables (*R* ^2^ = .17) and a small‐medium amount of variance in the affective response ratings to these Positive trials (*R* ^2^ = .14). A Pearson correlation confirmed the risk component was associated with positivity (*r* = .37, *p* = .004). The *negative* loadings of the PLS component on all 8 variables, together with an overall positive association with positive affect, are again consistent with the ECI hypothesis, but also with the positive attenuation view (Allen et al., 1999), of higher depression risk being associated with blunted emotional response to positive feedback.

Overall, the behavioral PLS regression results indicate that those with higher biopsychosocial risk profiles for depression derive both reduced negative and reduced positive affect from relevant socially evaluative feedback, relative to lower risk participants, in line with predictions based on the ECI hypothesis (Rottenberg et al., 2005).

### 3.2 FMRI results

In our previous paper (Dalgleish et al., 2017), across all participants we reported greater activation in the bilateral dACC and left AI when participants received negative compared with neutral social feedback, consistent with the wider social rejection literature (see (Eisenberger, 2012) for review) suggesting that this dACC‐AI matrix is implicated in the processing of “social pain”. However, we also found that these same regions were activated (along with the ventromedial prefrontal cortex (vmPFC) and ventral striatum bilaterally) when receiving positive (relative to neutral) social feedback. A conjunction analysis revealed that these activations in the dACC and AI were significantly present across both contrasts indicating a shared neural involvement in the processing of social rejection and inclusion information in these regions. Here, we wanted to examine the relationship between our multivariate depressive risk factors and neural activation to Negative and Positive (relative to Neutral) social feedback, both when considered together within one analysis in line these earlier results (Dalgleish et al., 2017), as well when considered separately.

### 3.3 Multivariate PLS correlation activation

In line with this, to assess the collective contribution of our set of biopsychosocial risk variables on the relevant activation maps, multivariate PLS correlation was first conducted across both Positive (minus Neutral) and Negative (minus Neutral) conditions of the Social Evaluation Task to assess any shared behavioral contribution, and thereafter on each of the two feedback contrasts separately.

#### 3.3.1 Shared behavioral contributions to Negative > Neutral AND Positive > Neutral feedback

**FIGURE 1:**
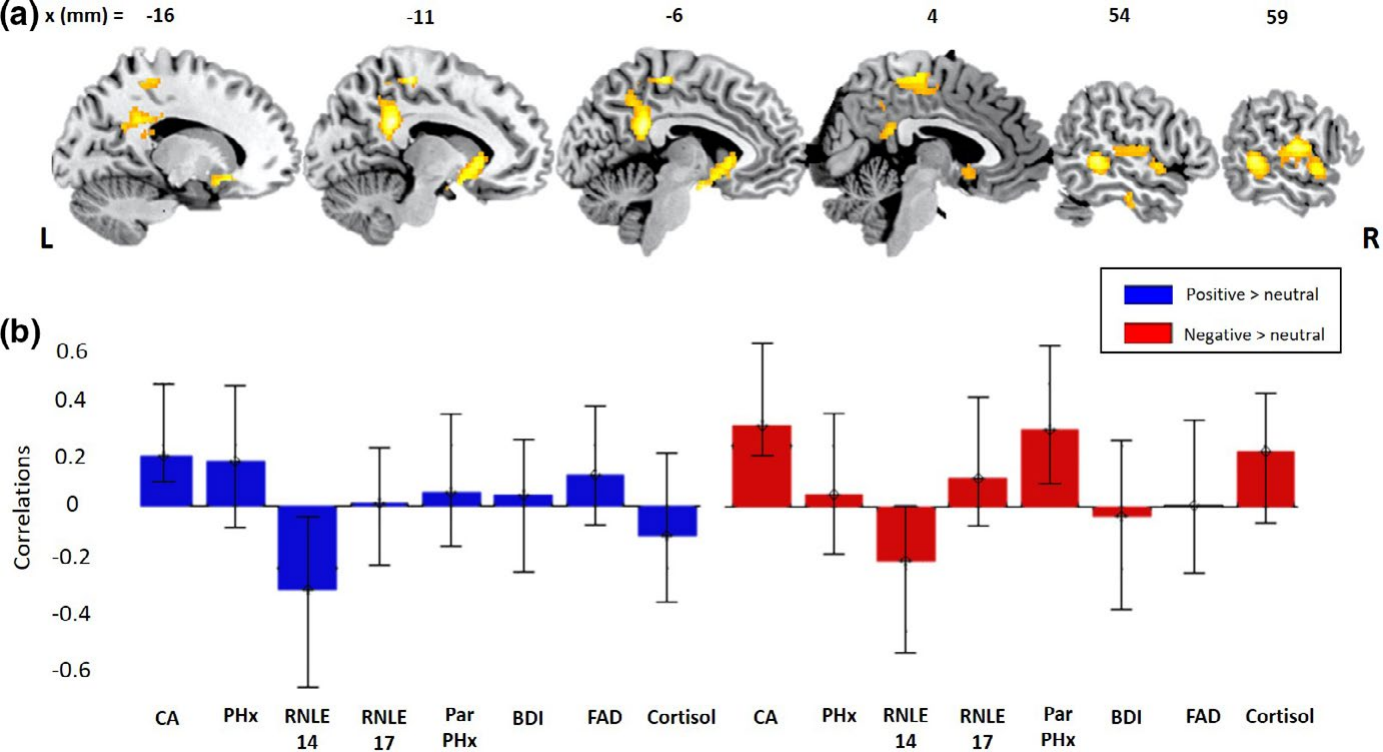
(a) Activated brain regions (R = Right. L = Left) and (b) behavioral correlations, with biopsychosocial risk variables from the PLS model examining neural responses to the Negative > Neutral AND Positive > Neutral feedback contrasts. BDI, Beck depression inventory; CA, childhood adversity; FAD, family assessment device; Par, parental; PHx, psychiatric history; RNLE, recent negative life events

A 2‐condition PLS model of the relationship between our collective risk variables and brain activity in response to both Negative > Neutral AND Positive > Neutral feedback conditions revealed one significant latent brain‐behavior pair, accounting for 30% of the variance (*d* = 124.2, permutation *p* = .003). Figure 1b shows the PLS behavioral saliences (transformed into correlations for ease of interpretation). Saliences are similar to the loadings in principal component analysis (PCA). The error bars show the 95% confidence intervals estimated from bootstrapping. This “correlation overview” graph shows that for the significant LV pair, there were stable correlations (i.e., confidence intervals not including zero) between the “brain scores” (i.e., the dot‐product of the brain LV saliences and the individual's imaging data, giving an overall summary of the brain data for each individual) (McIntosh and Lobaugh 2004) and presence of CA, in both the Positive AND Negative feedback conditions. For the remaining risk variables, the individual correlations were less robust as the confidence intervals included zero (in at least one of the two conditions), although it is important to note that these variables of course still contribute to the overall pattern of the brain‐behavior LV. The brain regions where this pattern was most reliably identified included a pattern of activation in the ventral striatum, posterior cingulate cortex, middle cingulate cortex, middle temporal gyrus and superior temporal gyrus (Figure 1a and Table 2). The data suggest a shared contribution of the presence of CA specifically (and of heightened risk generally) to patterns of increased activation in these regions across both Positive and Negative (minus Neutral) social feedback. Follow‐up univariate analysis revealed no significant activations.

**TABLE 2:** Activated brain regions associated with the Negative > Neutral AND Positive > Neutral feedback contrasts in the PLS model including the set of biopsychosocial risk variables

Bootstrap ratio	MNI X (mm)	MNI Y (mm)	MNI Z (mm)	Cluster size (voxels)	Cluster label
5.0506	18.0	−36.0	58.0	1,316	Middle cingulate cortex
4.9598	−10.0	−44.0	28.0	837	Posterior cingulate cortex
4.8309	−20.0	18.0	−12.0	738	Caudate
4.7258	64.0	−12.0	16.0	1,255	Postcentral gyrus
4.4612	56.0	−42.0	0.0	556	Middle temporal gyrus
4.4379	−60.0	−6.0	24.0	495	Postcentral gyrus
4.1325	36.0	−16.0	48.0	287	Precentral gyrus
4.0797	52.0	−20.0	−26.0	347	Inferior temporal gyrus

#### 3.3.2 Behavioral contribution to Negative > Neutral feedback

**FIGURE 2:**
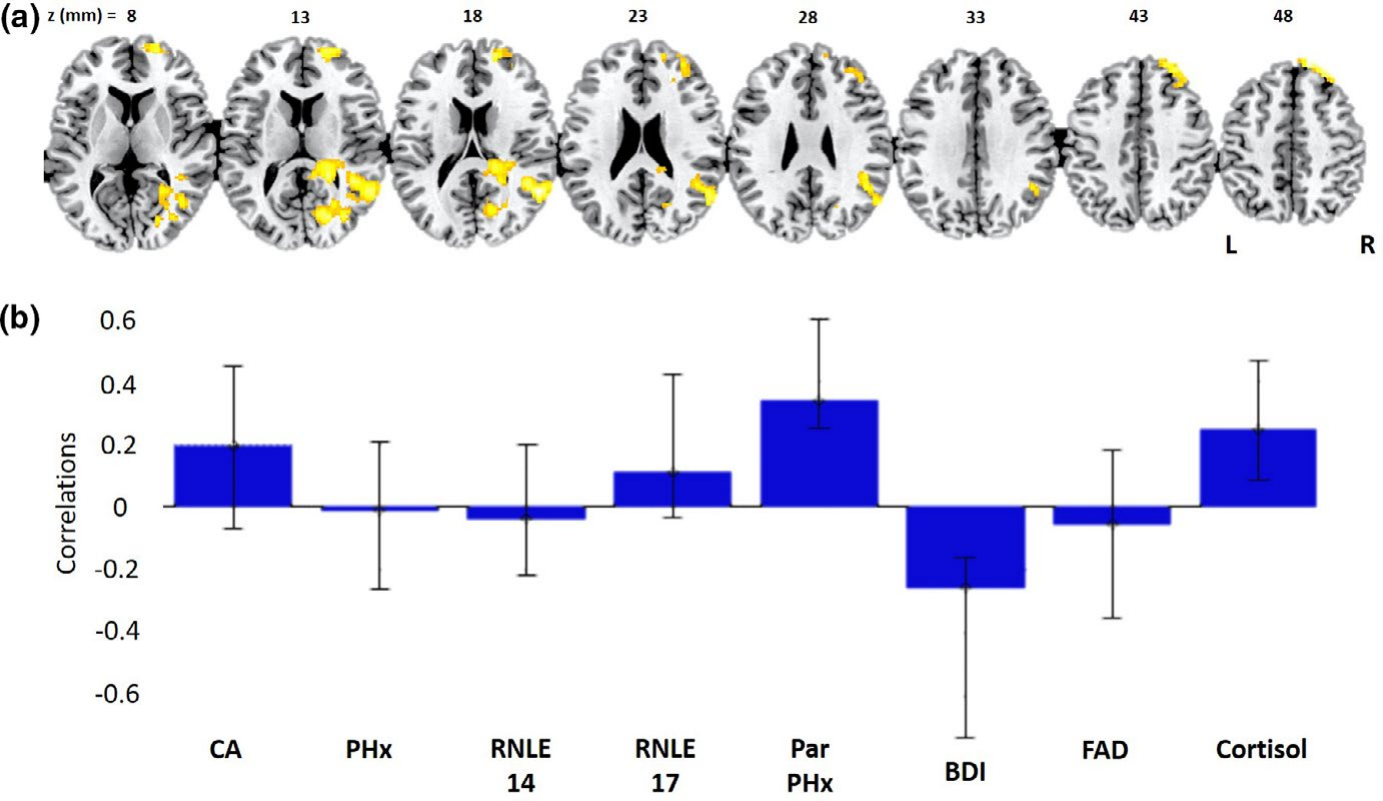
(a) Activated brain regions (R = Right. L = Left) and (b) behavioral correlations, with biopsychosocial risk variables from the PLS model examining neural responses to the Negative > Neutral feedback contrast. BDI, Becks depression inventory; CA, childhood adversity; FAD, family assessment device; Par, parental; PHx, psychiatric history; RNLE, recent negative life events

We next compiled a PLS model of the relationship between our collective risk variables and brain activity for the Negative > Neutral contrast considered alone. One significant latent brain–behavior pair (LV) was identified, which accounted for 33.4% of the covariance between activation in this contrast and our set of biopsychosocial risk variables (*d* = 97.1, permutation *p* = .037). Figure 2b shows the PLS behavioral saliences were robustly associated with three of the risk variables: parental psychopathology (*r* = .34), current BDI scores (*r* = −.26), and cortisol (*r* = .25). Parental psychopathology and cortisol showed positive associations, while BDI showed a negative association. The brain regions where this pattern was most reliably identified comprise the superior frontal gyrus, middle frontal gyrus, and posterior superior temporal lobe extending into the inferior parietal lobe and angular gyrus (see Figure 2a and Table 3). There were no significant active voxels for any of the univariate regression analyses.

**TABLE 3:** Activated brain regions associated with the Negative > Neutral Contrast in the PLS model including the set of biopsychosocial risk variables

Bootstrap Ratio	MNI X (mm)	MNI Y (mm)	MNI Z (mm)	Cluster size (voxels)	Cluster label
4.7941	54	−56	18	1,068	Superior temporal gyrus
4.7658	18	50	46	269	Superior frontal gyrus
4.6889	26	62	2	492	Middle frontal gyrus
4.3286	22	−76	14	925	Cuneus

#### 3.3.3 Behavioral contribution to Positive > Neutral feedback

Our PLS model revealed no significant brain‐behavior LV pair(s) for Positive > Neutral feedback trials considered alone, nor were there any significant univariate regression effects.

### 3.4 Sensitivity analysis: multivariate PLS activations excluding individuals with any psychiatric history

We subsequently ran the same three PLS analyses (negative and positive feedback together, negative alone, positive alone) as above on participants with no prior psychiatric history (*n* = 38) in order to test the sensitivity of the brain‐behavior relationships to prior mental illness (though it is worth reiterating that psychiatric history did not robustly contribute to the initial analysis, see Figures 1b and 2b for correlational overviews). Across all three analyses, a similar pattern of results was observed to those reported above (see Figures S3 and S4 and Tables S5 and S6). For the shared condition PLS, in line with our initial analysis only 1 LV pair was significant. This was robustly positively associated with CA across both conditions and involved the same brain regions (Figure S3 and Table S5). The Negative > Neutral feedback, in line with our initial analysis, indicated a robust negative association with BDI and a positive association with parental psychiatric history but now with the addition of a positive association with CA, and neurally encompassed the same brain regions as before (Figure S4 and Table S6). There was again no significant LV pair for the Positive > Neutral Feedback condition alone.

These results indicate that the findings including the whole sample were not significantly skewed by the inclusion of a minority of participants with a previous psychiatric history.

## 4 DISCUSSION

We investigated the relationship between a cluster of theoretically derived and empirically validated biopsychosocial depression risk variables and behavior and neural activations in a social evaluation task in a longitudinal population‐derived sample of late adolescents (Dalgleish et al., 2017). As hypothesized, and in line with the emotion context insensitivity (ECI) hypothesis of blunted emotional reactivity applied to the domain of depressive risk, the behavioral data revealed that risk was associated with both reduced negative affect following negative social feedback and reduced positive affect following positive social feedback. The behavioral results support the notion that adolescents characterized as higher in biopsychosocial risk of depression (and of psychopathology generally) display a similar profile of emotional reactivity to adults with MDD—specifically, reduced reactivity to *both* positive and negative social feedback—in line with the ECI hypothesis (Bylsma et al., 2008; Rottenberg et al., 2005). Importantly, this was the case even in those adolescents with no prior psychiatric history, suggesting that this blunted emotional reactivity style may predate the onset of any psychopathology, although of course the current data can only speak to risk variables. ECI is grounded in evolutionary theories of depression (Allen & Badcock, 2003; Beck & Bredemeier, 2016; Gilbert & Allan, 1998; Nesse, 2000), whereby it is proposed that dampened emotional reactivity is one component of a systemic disengagement from the environment to minimize continued activity which may be wasteful or dangerous in adverse situations (Bylsma et al., 2008).

In a previous study assessing cognitive reappraisal of emotion in the same sample of adolescents, we showed those with a history of CA (relative to those without) had an *enhanced* capacity to downregulate both positive and negative affect (Schweizer et al., 2016). We interpreted this as the CA environment serving as a practice ground to hone explicit emotion regulation skills. In the current study, a higher risk of depression predicted lower positive and negative affect following social feedback, in the absence of any emotion regulation instructions. The lack of uniqueness associated to any single variable reinforces our view that depressive risk is not related to any one single variable but is formed via the interplay between a constellation of biological, psychological, and social factors. The current results therefore extend these previous findings and support a more general notion of emotion attenuation associated with biopsychosocial risk.

In terms of neural activity, we identified latent brain‐behavior relationships associated with high biopsychosocial risk. First, in response to both cues of rejection (negative feedback) *and* inclusion (positive feedback), we observed a general pattern of increased cingulate, temporal, and striatal activity. Secondly, in response to *rejection only*, we observed a pattern of activity in ostensibly executive control‐ and emotion regulation‐related brain regions encompassing fronto‐parietal brain networks including the angular gyrus.

Research on brain reward‐region responsivity in association with biopsychosocial risk for depression has been mixed. Reward processing in those who have experienced early adversity has been accompanied by reduced activation of the ventral striatum in a number of studies (Goff et al., 2013; Hanson et al., 2016; Mehta et al., 2010), and this has been interpreted as an adaptive avoidant response during approach‐avoidance conflict situations (Teicher & Samson, 2016) conferring long‐term risk.. In contrast, and in line with the present findings of augmented activity in reward‐related brain networks as a function of risk, Dennison and colleagues (2016) reported increased striatal response, specifically in the left nucleus accumbens and putamen, while passively viewing positive relative to neutral social stimuli in a group of maltreated older adolescents relative to a control group with no history of maltreatment. Further, in a longitudinal community‐based study of adolescent girls, low parental warmth—a risk factor for subsequent MDD (Hipwell et al., 2008)—measured at age 11 was associated with increased striatal activity during reward anticipation measured at age 16 (Casement et al., 2014). Finally, the posterior cingulate and striatum show increases and decreases in response to up‐ and downregulation of socially driven emotions during neuroeconomic strategy games (Grecucci et al., 2013). The increased activation of these regions in response social evaluation in our study could therefore represent the downstream effects of the enhanced emotion regulation capabilities of the sample (Schweizer et al., 2016), potentially reflecting a putative resilience mechanism to social evaluation.

Our findings of increased activity in fronto‐parietal regions commonly associated with cognitive reappraisal of emotion (Buhle et al., 2014) and high‐order executive control (Burgess et al., 2007; Koechlin & Hyafil, 2007), in response to social rejection are in‐keeping with an ECI analysis whereby brain regions associated with cognitive control and emotion regulation are recruited to dampen emotion responses.

It is important to consider the relationship between our findings and notions of stress inoculation and resilience (Rutter, 2012). Evolutionary theorists (Allen & Badcock, 2003; Beck & Bredemeier, 2016; Gilbert & Allan, 1998; Nesse, 2000) argue that depressed *mood*, including the pervasive emotional insensitivity that we find here, is in fact an adaptive or resilient response to risks of social exclusion, illness, or threats to valued resources. Depressed mood serves to withdraw the beleaguered individual from potentially disadvantageous social disputes and shifts the focus toward repair and resource conservation. It is only when this systemic response becomes entrenched or chronic that *clinical* depression occurs. This suggests that those biopsychosocial factors that confer a greater risk for clinical depression will also likely confer a greater risk for pervasive depressed mood, including emotion context insensitivity, as a putative resilient response. In this context, then, risk and resilience are two sides of the same coin because depressed mood—an adaptive or resilient response—also places the individual at risk of clinical depression—a maladaptive response—if that mood state becomes entrenched. This interpretation is in line with the negative correlation we observed between depressive symptom severity (BDI) and the neural response pattern to negative social evaluation. Moreover, this complexity is supported by the results of our sensitivity analysis, which excluded participants with a prior mental health difficulty but revealed largely unchanged latent brain‐behaviour pairs relative to the whole sample. This sensitivity analysis subsample has navigated the period of mid‐adolescence associated with the greatest risk of onset of depression (and other disorders; (Spinhoven et al., 2010) without experiencing a psychiatric episode. For them, it therefore makes sense to characterise the relationship between elevated risk on our suite of biopsychosocial variables and emotion insensitivity as a putative marker of resilience.

It is important to note that, while a strength of the present study is the depth and extent of the assessment of depressive risk, there are nevertheless other depressive risk variables that may well contribute to the pattern of results we report here, which were not collected as part of the ROOTS protocol. Further studies would be welcome to assess the validity of our findings in similar population‐based cohorts, and to test the specificity of the brain‐behavior pairs with other neurocognitive profiles aside from psychosocial stress, such as in emotional regulation or cognitive flexibility paradigms. In addition, it should also be noted that we have a relatively small sample size (*N* = 56) to assess depressive risk across multiple parameters. This is however, why we opted to use the PLS method which has been validated for use in data such as this (Wold et al., 1984). Furthermore, the robustness of our neuroimaging analysis with 10,000 permutation tests and 10,00 bootstraps lend confidence to the validity of the brain‐behaviour relationships we observed.

In conclusion, this is the first study to our knowledge that has investigated the relationship between multivariate depressive risk and emotional response style, and latent brain‐behavior relationships of neurocognitive activation patterns during a social evaluation task. We provide tentative evidence to support the ECI hypothesis of emotional reactivity in adolescents at high risk of depression. The study is strengthened by the use of a population‐derived sample; however, in the absence of follow‐up data, we are unable to make firm inferences about the relationship of the current variables and later psychopathology.

## AUTHOR CONTRIBUTIONS

TD, IG, DM, and NW devised the study. NW and SS collected the data. JS, ML, and ALvH analyzed the data. JS and TD wrote the paper.

## FUNDING INFORMATION

All authors report no biomedical financial interests or potential conflict of interests.

### PEER REVIEW

The peer review history for this article is available at https://publons.com/publon/10.1002/brb3.2005.

## Supporting information

Data S1

## Data Availability

The datasets generated during and/or analyzed during the current study are available from the corresponding author on reasonable request.
