# Supplementary material for: How biopsychosocial depressive risk shapes behavioral and neural responses to social evaluation in adolescence
Source: Brain Behav. 2021 Mar 4;11(5):e02005. doi: 10.1002/brb3.2005 (PMC8119860; doi:10.1002/brb3.2005)
Supplement: Supplementary file 1 — Data S1 [file BRB3-11-e02005-s001.docx]

**Supplemental Material. How biopsychosocial depressive risk shapes behavioral and neural responses to social evaluation in adolescence.**

**Supplemental Methods**

**Participant Recruitment**

Participants were recruited through secondary schools as part of the ROOTS project (Goodyer et al., 2010). The aim of the ROOTS project was to determine the relative contributions of specific genetic, physiological, psychological and social variables to the overall risk for psychopathology emerging during adolescence. We sought to recruit a subsample of the ROOTS project cohort for the present study with a broad constellation of psychopathology risk. We consequently adopted a sampling strategy from the wider ROOTS cohort that was based on two risk indices – 5-HTTLPR genotype and presence/absence of childhood adversity – to provide a proxy of risk more broadly. To that end, we generated a list of all potential ROOTS participants who were eligible based on 5-HTTLPR genotyping and classification of CA using the Cambridge Early Experience Interview (CAMEEI) (see below). Subsequent to the study starting and prior to data analysis, the validity of 5-HTTLPR status as a risk factor for depression has been significantly undermined (Culverhouse et al., 2018) and we therefore decided to not include it in our final portfolio of biopsychosocial risk variables for depression.

The study coordinator (NW) attempted to contact 152 eligible participants, being either s/s or l/l homozygotes of the 5-HTTLPR allele, and either having (CA+) or not having (CA−) a history of childhood adversity (see below). Of these, 117 were contactable and 96 agreed to be asked the eligibility questions after being informed about the aims of the study. After a description of the study, participants underwent a brief phone screen to ascertain eligibility as defined by the inclusion/exclusion criteria. Eighty-seven participants proved eligible following this phone screen. If participants were eligible, invitation letters and information sheets were sent to interested participants. Following this, an initial assessment was arranged during which participants gave written informed consent. Of those eligible, 80 were recruited to the study. Of this number, 60 participants completed the scan session. One participant was removed due to imaging acquisition difficulties and we had doubts as to whether 3 participants fully believed in the veracity of the cover story for this particular task following the debriefing procedure. This left 56 participants with usable data as described in the present study. (See Figure S1).

**Assessment of childhood adversities (0–11 years) — the Cambridge Early Experiences Interview (CAMEEI)**

This semi-structured interview is conducted with the child's primary caregiver and records family-focused adverse life experiences, child's age at occurrence, duration, and an interviewer assessment of their practical impact on the daily life of the family (see ([Dunn et al., 2011](#_ENREF_4)) for more information). The current investigation used information covering the first eleven years of life to classify adolescents into those exposed (CA +, n = 26) and not exposed (CA −, n = 30) to early CA (See Table S2).The first eleven years was covered in order to make our groups comparable to the age limit for early maltreatment used in the maltreatment studies by Caspi and colleagues ([Caspi et al., 2002](#_ENREF_2); [Caspi et al., 2003](#_ENREF_3)). Exposure to an adverse family environment was defined as exposure to abuse (emotional, physical or sexual) and/or significant family discord; occasional physical violence, lack of affectionate warmth, or severe lack of communication between family members. In summary, amongst the 26 CA + participants, none reported sexual abuse. For physical abuse, 2 (8%) were classified as possibly being exposed and 1 (4%) was classified as yes/probably being exposed. For emotional abuse 4 (15%) were classified as yes/probably being exposed. All 26 had been exposed to moderate to severe inter-parental discord. Exposure was estimated to begin from birth with the duration estimated as ranging from 5 through to 56 months (mean 30.8 (s.d. 26.1) months).

**Description of participant psychiatric history**

Participants were longitudinally assessed for a past psychiatric diagnosis through their participation in the ROOTS study (using the Kiddie Schedule for Affective disorders and Schizophrenia for School-Age Children ([Kaufman et al., 1997](#_ENREF_6)) assessments). Retrospective inspection of the ROOTS data-set revealed that 18 participants (30%) (See Table S2) had prior DSM-IV diagnoses and these are reported in Table S1.

**Assessment of parental psychiatric history**

The MINI Mental State Examination ([Sheehan et al., 1998](#_ENREF_8)) was embedded within the CAMEEI assessment to assess parental mental illness during the participant's childhood. We also recorded disorder in biological parents prior to the birth of the participant and when living away from the family. Thresholds for inclusion were set very high with clear evidence of impairment essential for diagnosis. As most interviewees were mothers, the information on maternal mental health is the most reliable and valid and was corroborated using clinical notes. (See Table 1 & Table S2)

**Assessment of recent negative life events (RNLE) aged 13–14 & 16–17**

At ages 14 and 17, participants in the ROOTS cohort had completed a self-report measure of negative life events and difficulties [modified from ([Goodyer, Herbert, Tamplin, & Altham, 2000](#_ENREF_5))], occurring to them, their family or closest friends over the preceding 12 months. Participants were asked to date these experiences and rate their impact on themselves on a scale from 1 = very pleasant/happy to 5 = very unpleasant/sad/painful. If participants rated either 4 or 5 they were asked to indicate if they felt upset for longer than 2 weeks. From these ratings, two separate summed totals for positive and negative recent life events rated as occurring for longer than 2 weeks were derived. The negative events summed total were used here. At aged 14, the range was 0-3 events; 40 participants reported zero RNLE; 11 participants reported 1 RNLE; 4 participants reported 2 RNLE and 1 participant reported 3 RNLE. At aged 17, the range was 0-8 events; 30 participants reported zero RNLE; 19 participants reported 1 RNLE; 4 participants reported 2 RNLE; 2 participants reported 3 RNLE and 1 participant reported 8 RNLE. (See Table S2)

**Family Assessment Device — Global Functioning Subscale (FAD-GF)**

The FAD-GF ([Miller, Ryan, Keitner, Bishop, & Epstein, 2000](#_ENREF_7)) is a 12-item self-report scale measuring overall health/pathology of the family. Six items describe healthy functioning and the other six describe unhealthy functioning. Each item is rated on a 4 point Likert scale (4 = ‘strongly agree’, 3 = ‘agree’, 2 = ‘disagree’, 1 = ‘strongly disagree’). In ROOTS, positive items on this scale were reverse coded to measure overall negative family functioning. To facilitate interpretation in our full model below, we inverted these scores so that a high score reflects positive family functioning. The higher the score the worse the family functioning. (See Table S2)

**Assessment of current depressive symptoms at time of scanning**

The Beck’s Depression Inventory (BDI-II) ([Beck, Steer, Ball, & Ranieri, 1996](#_ENREF_1)) was used to assess current depressive symptoms in the two weeks prior to scanning. This assessment was administered on the day of the scanning session. (See Table S2 and Figure S2).

**Morning cortisol level**

Salivary cortisol samples were collected 45 min after waking within a week of baseline over 4 days. Cortisol assay was measured by ELISA on 20-μl samples of saliva without extraction (antibody; Cambio). Results are reported in ng/mL. Fifteen samples were either not adequate or not supplied leaving 41 samples for analysis. Missing data were generated using multiple imputation using the seven other risk variables as predictors in the model. Five multiple imputations were generated, and the mean of the pooled estimates were used in the final analysis. (See Table S2).

**Supplemental Results**

**Table S1.** Neuroimaging sample participant psychiatric history as diagnosed using the K-SADS.

| Group | Previous disorder |
| --- | --- |
|  |  |
| CA + | Previous NSSI, affective disorder(MDD), anxiety disorder (Panic disorder) |
| CA + | Previous anxiety disorder (Specific phobia) |
| CA + | Previous anxiety disorder (Specific phobia) |
| CA + | Previous NSSI, affective disorder (MDD), Anxiety disorder (Anxiety NOS) |
| CA + | Previous NSSI |
| CA + | Previous behavioral disorder (CD, ODD, ADHD) |
| CA + | Previous anxiety disorder (Panic disorder) |
| CA- | Previous affective disorder (MDD), anxiety disorder (Specific spider phobia) |
| CA- | Previous affective disorder (MDD), previous anxiety disorder (Panic attack) |
| CA- | Previous NSSI, previous MDD |
| CA- | Previous anxiety disorder (OCD & Panic attacks) |
| CA + | Previous NSSI, affective disorder (MDD), anxiety disorder (Panic disorder), alcohol abuse |
| CA + | Previous NSSI, affective disorder MDD, anxiety disorder (Panic disorder) |
| CA + | Previous NSSI |
| CA + | Previous eating disorder |
| CA + | Previous affective disorder (MDD) |
| CA- | Previous behavioral disorder (ADHD) |
| CA- | Previous alcohol abuse |

**_Abbreviations:_** _CA+ = Childhood Adversity; CA- = No Childhood Adversity; NSSI (Non-Suicidal Self Injury). MDD (Major Depressive Disorder). NOS (Not Otherwise Specified). CD (Conduct Disorder). ODD (Oppositional Defiant Disorder). OCD (Obsessive Compulsive Disorder). ADHD (Attentional Deficit Hyperactivity Disorder)._

**Table S2.** Descriptive statistics of the depressive risk variables used in the PLS-Regression.

|  | **Count** | **Mean** | **S.D.** | **Median** | **Range (minimum-maximum)** | **Interquartile Range** |
| --- | --- | --- | --- | --- | --- | --- |
| **BDI** | **n/a** | **4.52** | **5.19** | **3** | **0-27** | **5** |
| **Morning Cortisol Reactivity (mean(s.d.))** | **n/a** | **0.04** | **0.84** | **.02** | **-1.49 – 4.27** | **.6** |
| **FAD (mean(s.d.))** | **n/a** | **25.75** | **5.94** | **26** | **14-44** | **7.75** |
| **CA (+/-)** | **26/30** | **n/a** | **n/a** | **n/a** | **n/a** | **n/a** |
| **RNLE 14** | **0 = 40**  **1 = 11**  **2 = 4**  **3 = 1** | **n/a** | **n/a** | **0** | **0-3** | **1** |
| **RNLE 17** | **0 = 30**  **1 = 19**  **2 = 4**  **3 = 2**  **8 =1** | **n/a** | **n/a** | **0** | **0-8** | **1** |
| **Parental Psychopathology (+/-)** | **30/26** | **n/a** | **n/a** | **n/a** | **n/a** | **n/a** |
| **Previous Psychopathology (+/-)** | **18/38** | **n/a** | **n/a** | **n/a** | **n/a** | **n/a** |

**BDI = Becks Depression Inventory; FAD = Family Assessment Device; CA = Childhood Adversity; RNLE = Recent Negative Life Events; s.d. = standard deviation.**

**Table S3. Correlation Matrix of the Biopsychosocial risk variables.**

| **Correlations** | | | | | | | | | |
| --- | --- | --- | --- | --- | --- | --- | --- | --- | --- |
|  | | CA | PHx | RNLE 14 | RNLE 17 | Parental PHx | BDI | FAD | Cortisol |
| CA | Pearson Correlation | -- |  |  |  |  |  |  |  |
|  | N | 56 |  |  |  |  |  |  |  |
| PHx | Pearson Correlation | .279^*^ | -- |  |  |  |  |  |  |
|  | Sig. (1-tailed) | .019 |  |  |  |  |  |  |  |
|  | N | 56 | 56 |  |  |  |  |  |  |
| RNLE 14 | Pearson Correlation | -.137 | .088 | -- |  |  |  |  |  |
|  | Sig. (1-tailed) | .157 | .260 |  |  |  |  |  |  |
|  | N | 56 | 56 | 56 |  |  |  |  |  |
| RNLE 17 | Pearson Correlation | .171 | -.190 | .248^*^ | -- |  |  |  |  |
|  | Sig. (1-tailed) | .103 | .081 | .032 |  |  |  |  |  |
|  | N | 56 | 56 | 56 | 56 |  |  |  |  |
| Parental PHx | Pearson Correlation | .364^**^ | .257^*^ | -.016 | -.085 | -- |  |  |  |
|  | Sig. (1-tailed) | .003 | .028 | .452 | .266 |  |  |  |  |
|  | N | 56 | 56 | 56 | 56 | 56 |  |  |  |
| BDI | Pearson Correlation | .039 | .146 | .094 | -.048 | -.205 | -- |  |  |
|  | Sig. (1-tailed) | .389 | .141 | .244 | .363 | .064 |  |  |  |
|  | N | 56 | 56 | 56 | 56 | 56 | 56 |  |  |
| FAD | Pearson Correlation | .240^*^ | .049 | -.083 | .059 | .040 | .194 | -- |  |
|  | Sig. (1-tailed) | .037 | .361 | .271 | .333 | .386 | .076 |  |  |
|  | N | 56 | 56 | 56 | 56 | 56 | 56 | 56 |  |
| Cortisol | Pearson Correlation | .004 | -.180 | .076 | .303^*^ | .161 | -.151 | -.026 | -- |
|  | Sig. (1-tailed) | .488 | .092 | .288 | .012 | .118 | .134 | .425 |  |
|  | N | 56 | 56 | 56 | 56 | 56 | 56 | 56 | 56 |
| *. Correlation is significant at the 0.05 level (1-tailed). | | | | | | | | | |
| **. Correlation is significant at the 0.01 level (1-tailed). | | | | | | | | | |

Note: CA=Childhood Adversity; PHx = Psychiatric history; RNLE = Recent Negative Life Events; BDI = Becks Depression Inventory; FAD=Family Assessment Device.

**Table S4.** Biopsychosocial risk variable loadings of the PLS regression models on Positive and Negative response (minus Neutral) ratings

| Variable | Factor Loadings | |  | |  |
| --- | --- | --- | --- | --- | --- |
|  | Negative | Positive |  | |  |
|  |  |  | |  | |
| CA | .55 | -.44 |  | |  |
| PHx | -.07 | -.06 |  | |  |
| Parental PHx | .11 | -.39 |  | |  |
| RNLE14 | -.09 | -.22 |  | |  |
| RNLE17 | .41 | -.29 |  | |  |
| BDI | .44 | -.16 |  | |  |
| FAD | .56 | -.64 |  | |  |
| Cortisol | .22 | -.40 |  | |  |

CA=Childhood Adversity; PHx = Psychiatric history; RNLE = Recent Negative Life Events; BDI = Becks Depression Inventory; FAD=Family Assessment Device.

**Table S5.** Activated brain regions associated with the Negative > Neutral AND Positive > Neutral feedback contrasts in the PLS correlation model including the set of biopsychosocial risk variables in 38 participants with no psychiatric history.

| BSR | X(mm) | Y(mm) | Z(mm) | Cluster Size | Label |
| --- | --- | --- | --- | --- | --- |
| 4.9706 | 22 | 0 | 58 | 539 | Right Premotor Cortex |
| 4.7349 | 20 | 52 | 18 | 94 | Right Frontopolar Cortex |
| 4.7278 | -28 | 56 | 20 | 63 | Left Frontopolar Cortex |
| 4.4001 | -42 | 30 | 34 | 142 | Left Dorsolateral Prefrontal Cortex |
| 4. 3485 | -52 | -40 | 42 | 119 | Left Inferior Parietal Lobe |
| 4.2003 | 42 | 40 | 32 | 173 | Right Dorsolateral Prefrontal Cortex |
| 4.171 | 58 | -62 | 14 | 77 | Right Occipital Cortex |
| 4.1568 | 18 | -28 | 38 | 108 | Right Posterior Cingulate Cortex |
| 4.0133 | 26 | -80 | 8 | 84 | Right Visual Cortex |
| 3.9884 | 12 | -70 | -16 | 124 | Cerebellum |
| 3.86 | 36 | 38 | -6 | 114 | Right Inferior Frontal Gyrus |
| 3.7809 | 50 | -40 | 38 | 53 | Left Inferior Parietal Lobe |
| 3.6974 | -20 | -2 | 58 | 173 | Left Premotor Cortex |
| 3.6215 | 42 | 20 | 46 | 52 | Right Middle Frontal gyrus |
| 3.6195 | 34 | 12 | 4 | 60 | Right Insula |

**Table S6.** Activated brain regions associated with the Negative > Neutral feedback in the PLS correlation model including the set of biopsychosocial risk variables in 38 participants with no psychiatric history.

| BSR | X(mm) | Y(mm) | Z(mm) | Cluster Size | Label |
| --- | --- | --- | --- | --- | --- |
| 6.3359 | -36 | -22 | -18 | 2302 | Left Hippocampus |
| 5.4464 | 18 | -38 | 54 | 2603 | Right Inferior Parietal Lobe |
| 5.3417 | -12 | -46 | 24 | 3443 | Left Posterior Cingulate Cortex |
| 4.9771 | -18 | 32 | -2 | 1099 | Left Caudate |
| 4.7142 | 66 | -38 | -10 | 792 | Right Middle Temporal Gyrus |
| 4.5963 | 48 | -66 | 16 | 178 | Right Extrastriate Cortex |
| 4.3885 | -12 | -80 | 42 | 219 | Left Extrastriate Cortex |
| 4.3739 | -62 | -56 | 18 | 337 | Left Angular Gyrus |
| 4.2634 | -30 | 58 | 12 | 192 | Left Frontopolar Cortex |
| 4.1657 | 44 | 2 | -22 | 120 | Right Superior Temporal Gyrus |
| 4.1571 | 10 | 62 | 10 | 393 | Right Frontopolar Cortex |
| 3.9874 | 32 | -8 | 2 | 244 | Right Putamen |
| 3.9638 | 26 | 30 | 56 | 129 | Right Superior Frontal Lobe |
| 3.9394 | 34 | 34 | -4 | 82 | Right Insula |
| 3.9212 | 42 | 38 | 34 | 76 | Right Dorsolateral Prefrontal Cortex |
| 3.8928 | 20 | -82 | 46 | 187 | Right Visual Cortex |
| 3.5879 | -36 | -78 | 16 | 58 | Left Visual Cortex |
| 3.5275 | -18 | 46 | 44 | 183 | Left Superior Frontal Lobe |
| 3.5005 | -66 | -22 | 28 | 62 | Left Inferior Parietal Lobe |

**Figure S1.** Recruitment flow-chart

**
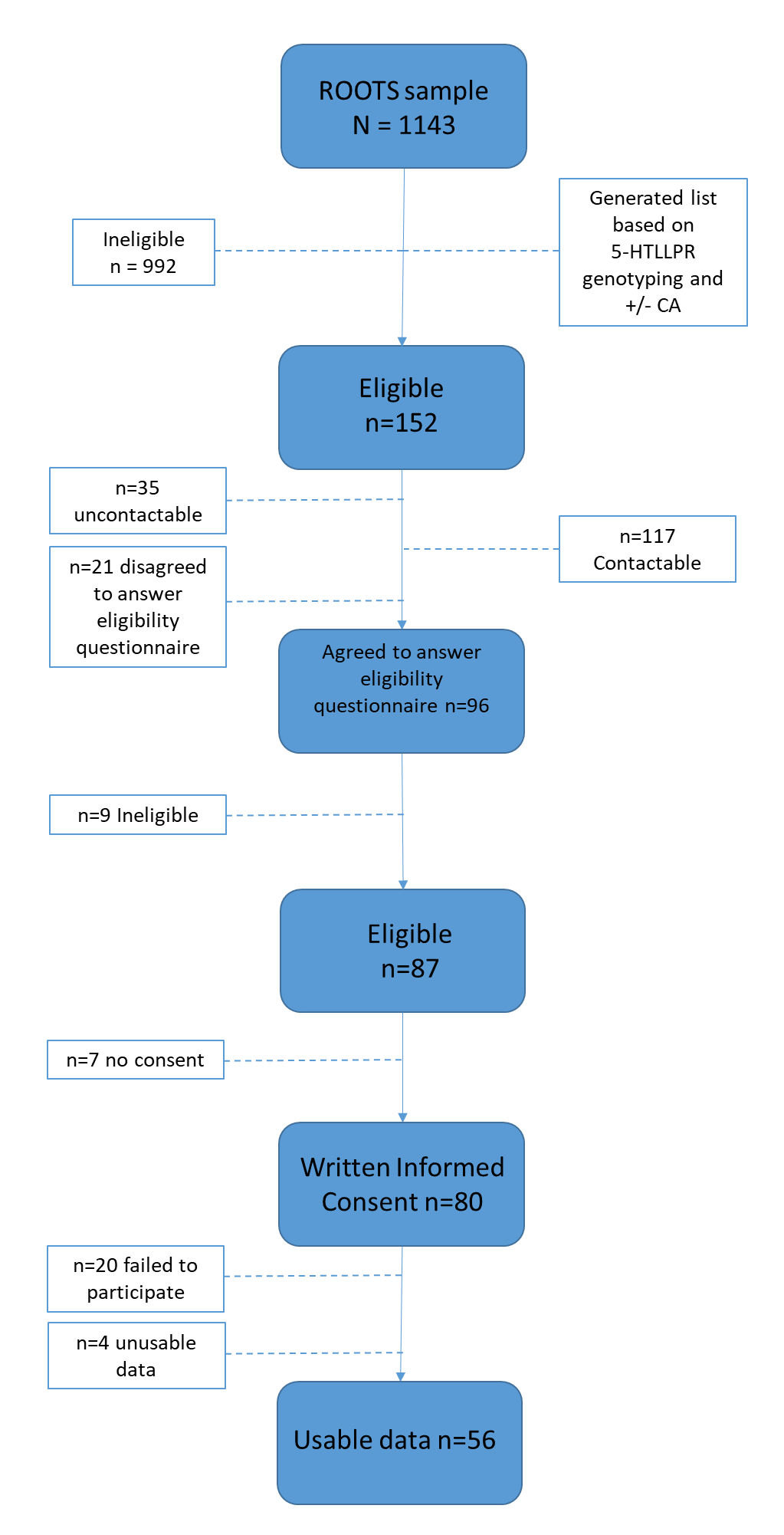
**

**Figure S2.** Histogram of depression scores as measured by the Beck Depression Inventory


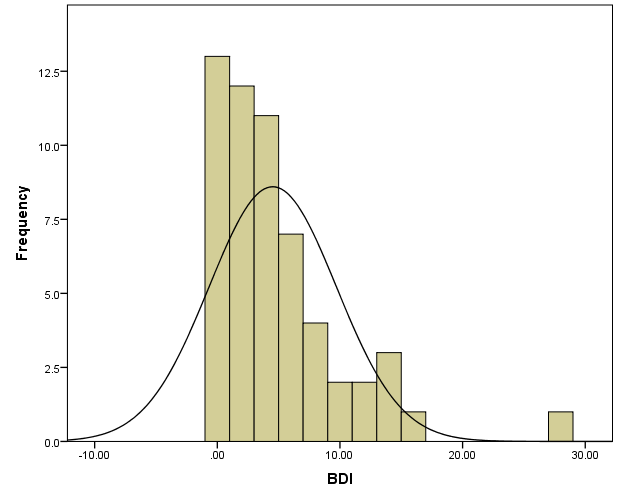


BDI; Becks Depression Inventory

**Figure S3.** (A) Activated brain regions and (B) behavioral correlations with biopsychosocial risk variables from the PLS correlation model examining neural responses to the Negative > Neutral AND Positive > Neutral feedback contrasts in 38 participants with no psychiatric history.


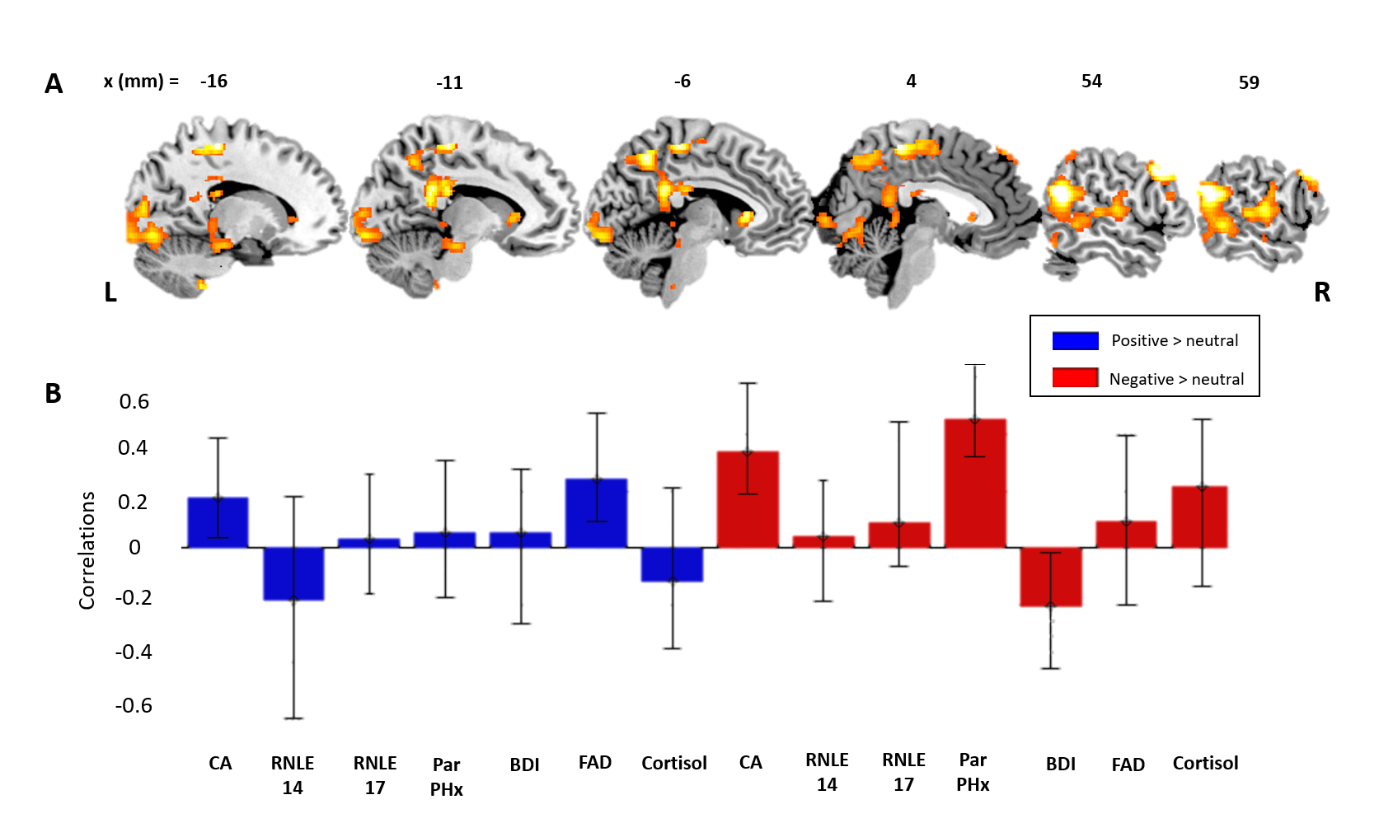


CA=Childhood Adversity; RNLE = Recent Negative Life Events; BDI = Becks Depression Inventory; FAD = Family Assessment Device; Par PHx = Parental Psychopathology.

**Figure S4.** (A) Activated brain regions and (B) behavioral correlations with biopsychosocial risk variables from the PLS correlation model examining neural responses to the Negative > Neutral feedback contrast in 38 participants with no psychiatric history.


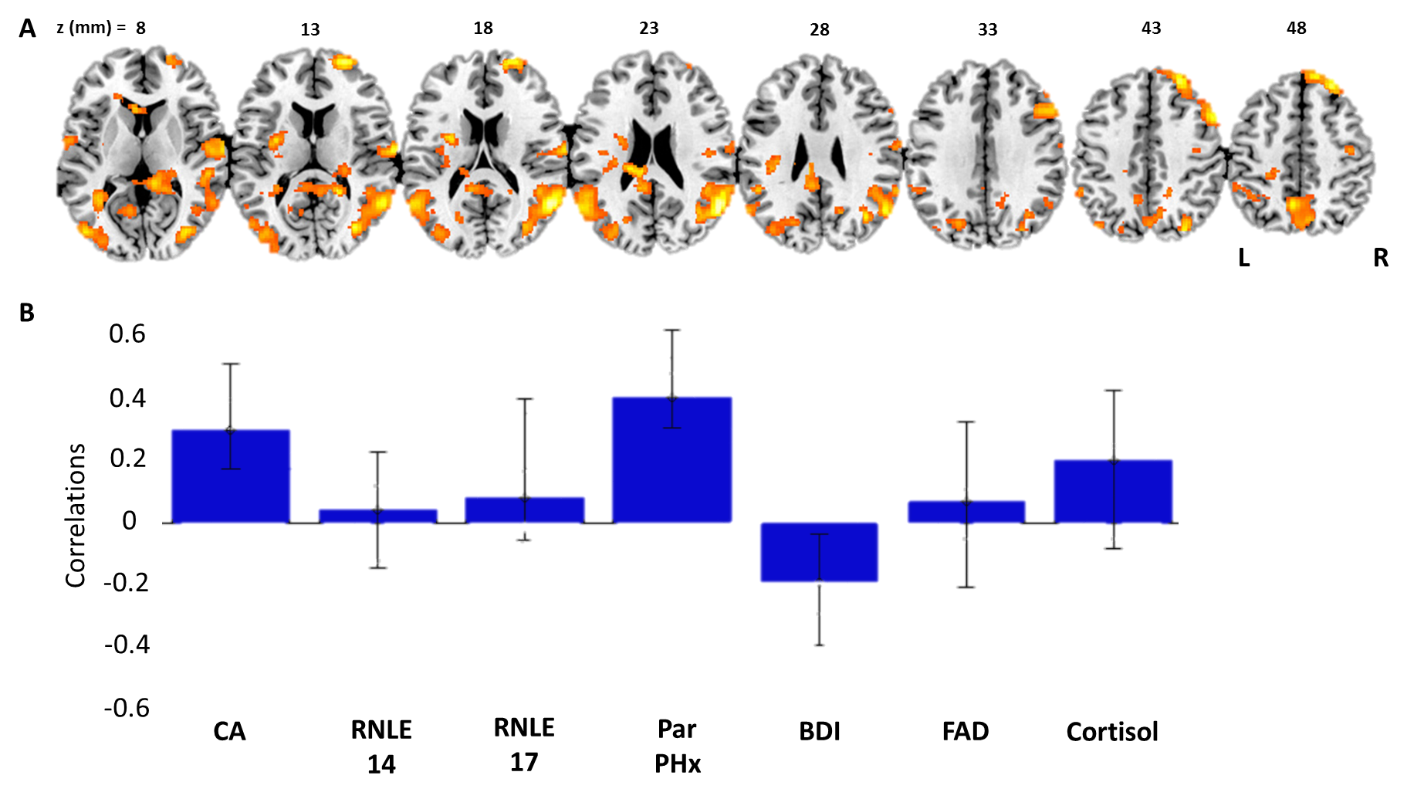


CA=Childhood Adversity; RNLE = Recent Negative Life Events; BDI = Becks Depression Inventory; FAD = Family Assessment Device; Par PHx = Parental Psychopathology.

**References**

Beck, A. T., Steer, R. A., Ball, R., & Ranieri, W. (1996). Comparison of Beck Depression Inventories -IA and -II in psychiatric outpatients. *J Pers Assess, 67*(3), 588-597. doi:10.1207/s15327752jpa6703_13

Caspi, A., McClay, J., Moffitt, T. E., Mill, J., Martin, J., Craig, I. W., . . . Poulton, R. (2002). Role of genotype in the cycle of violence in maltreated children. *Science, 297*(5582), 851-854. doi:10.1126/science.1072290

Caspi, A., Sugden, K., Moffitt, T. E., Taylor, A., Craig, I. W., Harrington, H., . . . Poulton, R. (2003). Influence of life stress on depression: moderation by a polymorphism in the 5-HTT gene. *Science, 301*(5631), 386-389. doi:10.1126/science.1083968

Dunn, V. J., Abbott, R. A., Croudace, T. J., Wilkinson, P., Jones, P. B., Herbert, J., & Goodyer, I. M. (2011). Profiles of family-focused adverse experiences through childhood and early adolescence: the ROOTS project a community investigation of adolescent mental health. *BMC Psychiatry, 11*, 109. doi:10.1186/1471-244X-11-109

Goodyer, I. M., Herbert, J., Tamplin, A., & Altham, P. M. (2000). Recent life events, cortisol, dehydroepiandrosterone and the onset of major depression in high-risk adolescents. *Br J Psychiatry, 177*, 499-504.

Kaufman, J., Birmaher, B., Brent, D., Rao, U., Flynn, C., Moreci, P., . . . Ryan, N. (1997). Schedule for Affective Disorders and Schizophrenia for School-Age Children-Present and Lifetime Version (K-SADS-PL): initial reliability and validity data. *J Am Acad Child Adolesc Psychiatry, 36*(7), 980-988. doi:10.1097/00004583-199707000-00021

Miller, I. W., Ryan, C. E., Keitner, G. I., Bishop, D. S., & Epstein, N. B. (2000). The McMaster Approach to Families: theory, assessment, treatment and research. *J. Fam. Ther., 22*(2000), 168-189.

Sheehan, D. V., Lecrubier, Y., Sheehan, K. H., Amorim, P., Janavs, J., Weiller, E., . . . Dunbar, G. C. (1998). The Mini-International Neuropsychiatric Interview (M.I.N.I.): the development and validation of a structured diagnostic psychiatric interview for DSM-IV and ICD-10. *J Clin Psychiatry, 59 Suppl 20*, 22-33;quiz 34-57.
